# The promise of stem cell-derived islet replacement therapy

**DOI:** 10.1007/s00125-020-05367-2

**Published:** 2021-01-16

**Authors:** Douglas Melton

**Affiliations:** Department of Stem Cell and Regenerative Biology, Harvard Stem Cell Institute, Harvard College and Medical School, Cambridge, MA USA

**Keywords:** Beta cells, ES cells, Immune protection, In vitro differentiation, iPS cells, Maturation, Review, Stem cells, Transplantation, Type 1 diabetes

## Abstract

Present-day treatments for people that are insulin dependent require multiple insulin injections, sometimes with an insulin pump, coupled with regular blood glucose monitoring. The availability of modified insulins, each with peaks of activity at varying times, has improved diabetes management. On the other hand, there have been impressive results leading to insulin independence by transplantation of cadaveric islets coupled with immune suppression. This review focuses on the possibility of treating diabetes with cellular transplants, specifically with the use of pluripotent stem cells, to produce a virtually unlimited and uniform supply of human islet-like clusters by directed differentiation. Prospects for improving the in vitro differentiation of human endocrine cells for the study of endocrine function and their possible clinical uses are also discussed.

**Supplementary Information:**

The online version of this article (10.1007/s00125-020-05367-2) contains a slideset of the figures for download.

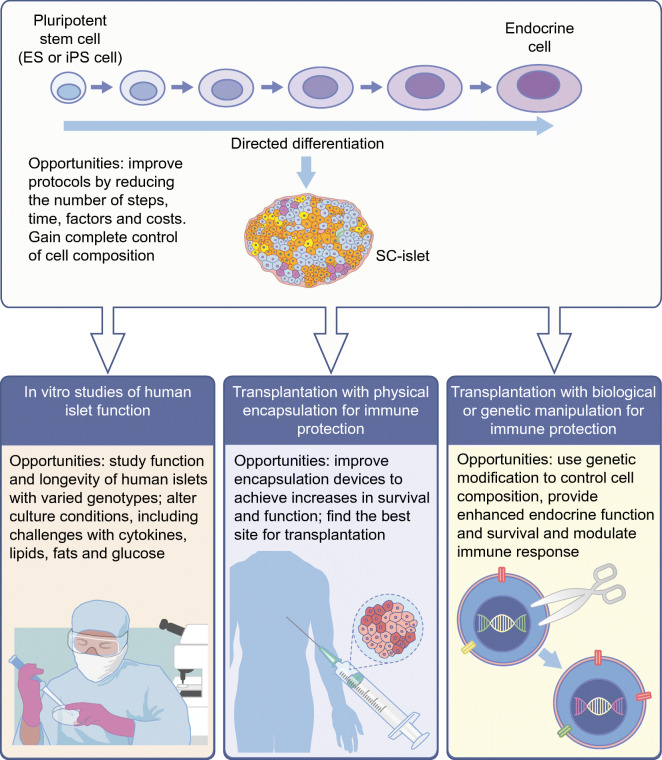

## Introduction

The discovery of insulin in the 1920s transformed the lives of insulin-dependent people with diabetes. This discovery is the subject of another review in this volume [1], but suffice it to say that the identification of insulin, its purification from various animal sources and its eventual production from the cloned human gene, made it possible to provide human insulins to people with diabetes. The advent of fast- and slow-acting insulins, and insulin pumps coupled with continuous glucose monitors (CGMs) defines some of the important innovations in present-day treatments. Despite these impressive advances, patients want and need more; the near-constant burden of monitoring blood glucose levels, insulin pumps, diet and exercise weighs heavily on patients and their families. And despite this considerable attention and the associated cost, our treatments still lead to comorbidities and a life with social and health burdens. Simply put, individuals with diabetes and their families live every day and night coping with the disease. Here, I discuss the possibility of a different approach.

The aim of CGMs and insulin pumps is to replace the absence of beta cells. Pancreatic beta cells evolved over millions of years to measure blood glucose levels accurately and quickly and deliver just the right amount of insulin. The beta cell reads glucose levels every millisecond and the insulin secretion by a cohort of beta cells is exquisitely coordinated. Replicating this biology is a challenge for CGMs (which read glucose levels every 5–15 min) and insulin pumps, and the gaps go a long way to explain why present-day treatments are not a cure and result in diabetic complications.

## Using living cells instead of machines to control blood glucose

Instead of using machines to recapitulate what the beta cell does, a more natural and effective solution may be to make human beta cells and transplant them as a regenerative medicine. Transplanting beta cells is not a new idea; cadaveric islets have been transplanted effectively for decades [2]. That procedure does require living with life-long immunosuppressants, but transplantation of cadaveric islets into the portal vein has demonstrated the power of cell replacement for controlling blood glucose levels. Cadaveric islets read blood glucose levels and deliver insulin so effectively that some patients self-report this therapy as a life-changing operation, making them feel ‘non-diabetic’. While insulin independence is not achieved in all cases, and typically lasts for 5–6 years, the results unequivocally demonstrate the effectiveness of islet cell replacement therapy.

Since cadaveric islets are not available in a sufficient supply nor quality to meet the needs of the millions of individuals in need of insulin therapy, other sources of human beta cells have been explored for decades. Researchers also considered the possibility of an endogenous stem cell, similar to blood stem cells, that could replenish missing beta cells. It is now largely agreed that no such adult stem cell for beta cells exists and, instead, beta cell mass is maintained by a very slow rate of self-replication [3]. It has proven to be quite difficult to increase the rate of endogenous beta cell replication, but recent work with chemical screens identified stimulants, such as harmine, that show promise. [4]. In addition, while there is evidence in rodents for the conversion of other adjacent cell types into beta cells [5], at this time there does not seem to be a straightforward way to transdifferentiate alpha cells or ductal cells into a sufficient mass of beta cells to treat diabetes. For example, transdifferentiation of exocrine cells into functional beta cells [6] requires ectopic expression of multiple transcription factors and this is not easily achieved in vivo with present technologies.

With the discovery of human pluripotent stem cells (both embryonic stem (ES) and induced pluripotent stem [iPS] cells), it became clear that one could, in principle, use their virtually unlimited potential for division and differentiation to solve the problem of making more beta cells. Setting aside the regulatory and ethical issues in obtaining these human cell types, the challenge taken on by several laboratories and companies became directing the differentiation of pluripotent cells into beta cells. Several groups [7–11] showed that it was possible to make cells that expressed several key beta cell genes, including insulin, in vitro. In most cases, the approach involved a stepwise differentiation protocol wherein the ES/iPS cells are first directed to definitive endoderm, followed, in turn, by gut development, pancreatic progenitors, endocrine/islet progenitors and, finally, beta-like cell differentiation. This work was guided by studies of pancreatic development in frogs, zebrafish and mice [12], and the resulting insights were supplemented with empirical screening, i.e. testing the ability of various small molecules and growth factors in the media to drive differentiation.

Demonstrating that human ES cells could make immature beta cells, cells with the potential to become functional beta cells (defined as cells that respond to repeated glucose challenges by secreting insulin, not cells that simply make and secrete insulin) was an important step. Months after transplantation in vivo, ES cell-derived beta cells were able to advance or complete their differentiation, further mature, and become glucose responsive, as evidenced by the successful treatment of pre-existing diabetes in immune-deficient mice following transplantation [13, 14]. These findings showed that the in vitro differentiation of ES cells could be advanced to a progenitor stage, an important step toward the end goal of making functional human beta cells.

## Using stem cells to produce functional beta cells in vitro

**Fig. 1:**
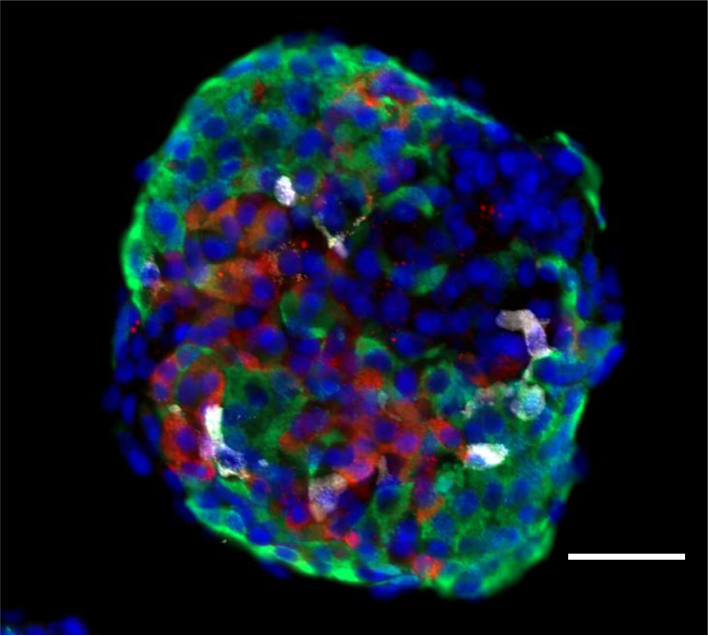
An SC-islet produced from human embryonic stem cells by a six-step differentiation procedure. Image of a representative SC-islet, stained and sectioned, showing the mixed population of cells present at the end of the differentiation protocol [23]. The protocol used was a six-step procedure of media and signalling factor changes, taking place in a spinner flask. Spherical SC-islets contain about 5000–10,000 cells. Immunohistochemistry highlights three different endocrine cell types: beta cells (green; stained for C-peptide), alpha cells (red; stained for glucagon) and delta cells (white; stained for somatostatin). Note there are other cells in the cluster that did not stain for any of these three hormones. Nuclei are stained blue with DAPI. Scale bar, 100 μm. This figure is available as part of a downloadable slideset

While it was clearly possible to produce cells that made insulin, it remained a challenge to produce functional beta cells in vitro, cells that responded to multiple glucose challenges by secreting insulin without requiring further differentiation and development in vivo following transplantation. Using multiple screening regimens and repeated testing, the goal of making physiologically functional cells in vitro was achieved in 2014 (see Fig. 1) [15]. Human ES and iPS cells were differentiated into functional beta cells by a six-step procedure, taking several weeks and using a combination of 2–5 differentiation signals at each of the six steps. While the combination and concentrations of factors and timing all played a role in this achievement, among the key advances were the use of gamma secretase inhibitor, activin receptor-like kinase 5 inhibitor (ALK5i) II and T_3_ stimulation at the final stages [15]. We shared these advances prior to publication with the biotech company Betalogics who then worked with another laboratory to reproduce our findings. Their paper [16] provided an independent verification, confirming that our protocol produced glucose-responsive beta cells entirely in vitro. This advance has been extended by others to other pluripotent cell lines, with various improvements [17, 18].

It is notable that the production of functional human beta cells in vitro occurs in the absence of natural cell contacts from mesenchyme, endothelia and other cells that provide instructive signals to pancreatic epithelia during development (e.g. [19–21]). Presumably, many of those endogenous signals are replaced by the factors added in vitro, but it is also possible that intercellular inductive signalling occurs among cells in the differentiation protocol [21, 22].

The in vitro differentiation protocols that have subsequently been developed are largely similar in that they all produce a mixture of cell types at the final stage (i.e. they do not produce only beta cells). It is also worth noting that each starting cell line requires some optimisation, but both ES and iPS cell lines can be successfully differentiated.

A more complete characterisation of the cell types produced by in vitro differentiation of ES or iPS cells has come from single-cell RNA sequencing (RNA-Seq). A gene expression profile of the cell types present in normal human islets was provided by single-cell RNA-Seq [23], confirming years of histological analyses of human islets and setting the goal for the cell composition of cells to be made using in vitro differentiation protocols. Veres et al. applied single-cell RNA-Seq to examine the cells present in stem cell-derived islets (SC-islets) and found fewer beta and alpha cells than are present in cadaveric islets, some exocrine cells, undifferentiated cells and enterochromaffin cells [24]. The latter was unexpected as these cells are normally in the gut. This analysis also demonstrated the remarkable stability of the differentiated cells produced: differentiated SC-islet cells maintain their phenotype in vitro for more than 5 weeks in the absence of any further differentiation signals. These findings further highlight the differences between progenitor cells [13] and more differentiated SC-islet beta cells that are capable of repeated responses to glucose stimulation in vitro [15].

**Fig. 2:**
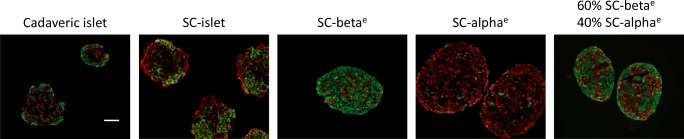
Creating SC-islets of defined cell composition. Cell surface markers on beta cells (CD49a) and alpha cells (CD26) were conjugated with antibodies bound to iron and then magnetically purified. Representative examples show staining of human cadaveric islets, SC-islets at the end of the six-step differentiation protocol, and SC-islets enriched with beta (SC-beta^e^) and alpha (SC-alpha^e^) cells by antibody purification and reassembled into islet-like clusters. Note the SC-islets enriched for beta and alpha cells by antibody purification are nearly, but not completely, pure. Some enriched SC-islets were recombined to form a specific ratio of beta:alpha cells (60% beta cells:40% alpha cells), as shown. Green, C-peptide (beta cells); red, glucagon (alpha cells). All clusters contained cells that did not stain for either C-peptide or glucagon and are other endocrine or non-endocrine cells. Scale bar, 50 μm. This figure is available as part of a downloadable slideset

Single-cell RNA-Seq also identified mRNAs encoding cell-type-specific surface proteins, CD49a for beta cells and CD26 for alpha cells. This makes it possible to greatly enrich alpha and beta cells (Fig. 2), enabling us to produce SC-islets with defined proportions of endocrine cell types. Similarly, expression of glycoprotein 2 (GP2) can also be used to obtain beta cell enriched cellular preparations [25, 26].

SC-islets thus appear to have the cell types needed to provide physiological function. Future efforts will likely focus on adjusting the proportions of endocrine cells (primarily alpha, beta and delta cells), ridding the final product of undifferentiated and unnecessary cells, as well as scaling the procedures in bioreactors and reducing variation in these complex multi-step protocols.

## Differences between SC-islets and adult cadaveric islets

While making beta cells that respond to successive glucose challenges represents a significant advance, the final product is not an exact replica of a human islet, neither in cell composition nor in full function. For example, the beta cells within SC-islets differ from cadaveric beta cells in terms of RNA transcripts encoding urocortin-3 (UCN3), islet amyloid polypeptide (IAPP), and oestrogen-related receptor gamma (ERRγ) [27, 28]. There are also differences in epigenetic modifications, including DNA methylation and histone modifications [29, 30]. Moreover, the circadian clocks of the individual cells in SC-islets are not synchronised, as they are in cadaveric islets [30].

During normal mouse development, there is a metabolic switch in the fetus, from a constitutive amino acid-stimulated insulin secretion to a postnatal, periodic glucose-stimulated insulin secretion. This maturation to oxidative metabolism correlates with a change from mammalian target of rapamycin complex 1 (mTORC1) to AMP-activated protein kinase (AMPK) signalling [31, 32], and this switch to AMPK signalling has also been shown to be correlated with the increased mitochondrial number in beta cells after birth [31]. Inhibiting mTORC1 with rapamycin in vitro drives SC-islets to a more mature phenotype [32]. Similarly, entraining the circadian clock in vitro further enhances and coordinates SC-islet function in vitro [30]. Moreover, ectopic expression of ERRγ via an adenoviral vector advances the differentiation of SC-islets for some cell lines [28]. All of these additional steps that fine tune beta cell function evidently take place following SC-islet transplantation, perhaps owing to endothelial signals or paracrine factors that are supplied following vascularisation.

A biochemical study of glucose metabolism in SC-islets points to a specific enzymatic block that may explain why SC-islet beta cells have a reduced magnitude of glucose-stimulated insulin secretion in vitro, as compared with those of cadaveric islets. Using mass spectrometry to analyse metabolites following a glucose challenge, reduced replenishment of tricarboxylic acid cycle (TCA) intermediates in the mitochondria of SC-islet beta cells was revealed [33]. This study identified a bottleneck in the glycolytic pathway at the stage in which glyceraldehyde 3-phosphate dehydrogenase and phosphoglycerate kinase act. Bypassing this metabolic bottleneck in vitro results in a robust, bi-phasic insulin release that is identical in magnitude to that in functionally mature human islets.

It is not clear how the changes in enzymatic and mitochondrial activity, transcription, epigenetic modifications and the circadian rhythm relate to one another in terms of their relative timing or control of one another. Importantly, SC-islets do achieve further maturation in vivo, post transplantation, and it may be unrealistic to expect their in vitro function to mimic cadaveric islets in every respect because the latter have had quite a different developmental history and contain many other kinds of non-endocrine cells, including mesodermal cells.

The studies reviewed above demonstrate that it is possible to use undifferentiated pluripotent ES or iPS cells to produce functional human pancreatic islet cells in vitro. Moreover, it is remarkable that ES/iPS cells develop so well in vitro, in the absence of all the normal milieu and three-dimensional cell interactions that normally orchestrate pancreatic islet development [34]. One can anticipate improvements in the differentiation protocols available by reducing the number of steps and factors employed, substituting small molecules (which may be expensive) for growth factors, and gaining more complete mastery over the differentiation so as to make islets with a defined composition of endocrine cell types. In addition, it will certainly help to more thoroughly study and reproduce the extracellular matrix (ECM), innervation and endothelial signals that occur in vivo for in vitro studies [35, 36]. The availability of SC-islets in virtually unlimited quantities, derived from different genetic backgrounds, will likely lead to informative studies on islet cell maturation and longevity, insulin resistance and the stress of exposure to high glucose and lipids [37], all of which are difficult to study in human participants.

As noted above, differentiation protocols can result in a final product that contains ‘unwanted’ cells, such as enterochromaffin cells or, in some instances, undifferentiated cells that can form other cell types or cysts following transplantation. Modifying the differentiation signals, using genetic modifications or the use of ‘suicide’ genes [38] may prove effective in ensuring that the final product is free of potentially harmful cells and contains the optimal cellular composition.

## Awaiting results from clinical trials

For clinical applications, present efforts are focused on treatment of type 1 diabetes, which requires some form of immune protection. The SC-islets will be transplanted into a foreign environment, not back into the pancreas. Since insulin injections are effective at many sites, the principal considerations are an adequate blood supply and ease of transplantation. Explorations of subcutaneous and intraperitoneal sites are presently being investigated as potential transplant environments; as of yet, it is not known which site offers the best vascular support for long-term survival and function. The liver is effective for the transplantation of cadaveric islets but the kidney capsule has been the most commonly used site for cadaveric islet transplantation in pre-clinical studies. In addition to choosing a site for SC-islet transplantation, the dose or number of SC-islets required to achieve metabolic control is not yet known. Based on inferences from cadaveric islet transplantation, it is generally believed that 1–5 × 10^8^ beta cells will be sufficient. However, it is not yet known how many alpha, delta or other cells are required to improve beta cell survival and function. This issue is directly related to improving the stem cell differentiation protocols to increase the percentage of beta cells in the final product.

If the cost of producing autologous iPS cells and differentiating each individual line into functional islets can be reduced, providing autologous SC-islets to insulin-dependent individuals with type 2 diabetes may also be feasible. It is not known how long transplants of autologous beta cells would effectively provide insulin in the environment that led to insulin dependence.

In addition to better understand the optimal dosage and cell composition of SC-islets, the challenge of providing an adequate blood supply and avoiding or reducing immune rejection is receiving considerable attention. For individuals with type 1 diabetes, the most advanced SC-islets prepared for clinical trials are produced from ES cells, which means that there will be both an autoimmune and alloimmune reaction to the transplanted cells. Furthermore, even if autologous iPS cells were used to make SC-islets for individuals with type 1 diabetes, a vigorous autoimmune response is expected following transplantation.

Several approaches are being explored to attend to the immune responses to and blood supply for transplants. First, encapsulating the SC-islets in a device that allows for nutrients and insulin to efficiently cross the membrane while preventing cells from trespassing is an attractive and near-term possibility. ViaCyte has ongoing clinical trials with progenitor cells placed in an encapsulation device. Semma/Vertex has also announced their intention for clinical trials using functional SC-islets and a different propriety encapsulation device. Methods for encapsulation with alginate derivates and novel biomembranes remains an area of active investigation and have been reviewed elsewhere [39].

**Fig. 3:**
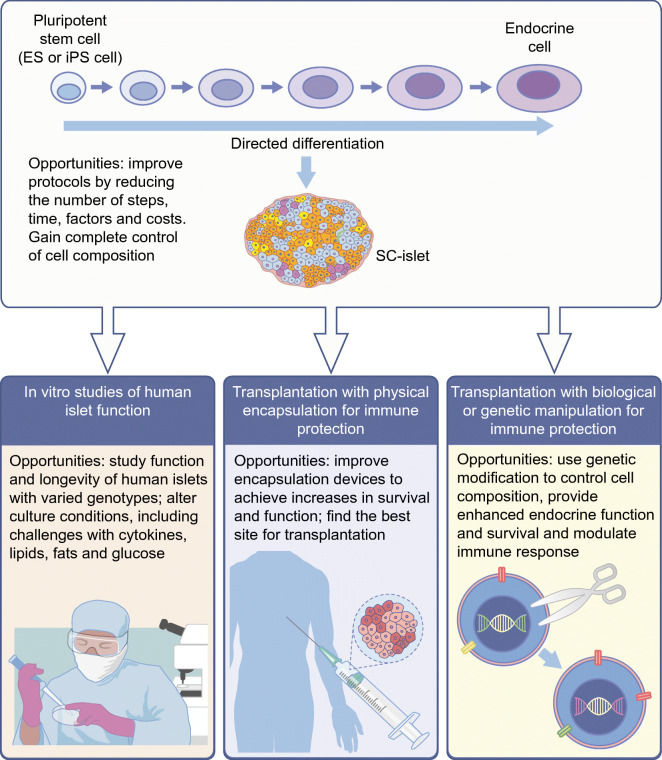
Possible uses and advances for in vitro studies and clinical applications of SC-islets. Pluripotent stem cells can be differentiated in vitro to produce SC-islets. SC-islets have the potential to be used in vitro, for example, to study human islet function, and clinically, to improve glucose management in insulin-dependent individuals by transplantation. Although many advances have been made in the development and transplantation of SC-islets, there are still areas for improvement, including improvements in the differentiation protocol for SC-islet generation, development of encapsulation devices to evade immunity against transplanted islets and genetic modification of SC-islets to enhance function and survival. This figure is available as part of a downloadable slideset

Biological solutions to immune rejection are also being explored, both at the local and systemic levels. Modifying the patient’s immune system is one approach, and advances in cancer immunotherapy provide important clues as to how cells can evade immune elimination (e.g. [40]). Effective interventions may use antibodies to block the immune reaction, and manipulation of specific populations of regulatory T (Treg) cells is a promising approach, especially as it may be possible to provide more specificity by identifying the immune T cells that are diabetogenic. A complementary approach focuses on the local immune reaction, genetically modifying the SC-islets so that they might evade immune attack [41, 42] or, in the ideal case, induce tolerance. However, this is a complex problem involving many immune cell types and many genes. One can be optimistic that the advances in genetic modifications and new assays for immune interactions with SC-islets [43] might allow for naked cell transplants using genetically modified SC-islets (Fig. 3) [44]. Of course, genetically modified cells carry a potential risk. It will be important to demonstrate that, in addition to the beneficial effects, the genetic modifications do not produce cells that form tumours or are otherwise harmful.

## Conclusion

In summary, there are fundamentally two challenges to treating or curing type 1 diabetes; the first is the absence of pancreatic beta cells. This has been treated for 100 years by insulin injection, which has more recently been coupled with blood glucose monitors. Making functional human islets from stem cells is now sufficiently advanced to contend that the challenge of producing islets from stem cells has been essentially overcome. There will be improvements in the manufacture of SC-islets, such as altering the proportion and types of cells produced, removing unwanted cells from the final product and scaling the manufacturing, but the challenge of making functional human beta cells from pluripotent stem cells has been achieved. The second problem is the persistence of an autoimmune attack. While autoimmunity in type 1 diabetes may not be the most robust and vigorous immunological reaction, it is certainly sufficient to eliminate enough beta cells to cause insulin dependence. Whether mitigated by physical encapsulation or biological interventions, the challenge of protecting transplanted SC-islets from immune reactions is surely worthy of collaborative and coordinated attention.

## Supplementary Information

Slideset of figures (PPTX 1.01 mb)

## Abbreviations

AMPK: AMP-activated protein kinase
CGM: Continuous glucose monitor
ERRγ: Oestrogen-related receptor gamma
ES: Embryonic stem (cells)
iPS: Induced pluripotent stem (cells)
mTORC1: Mammalian target of rapamycin complex 1
RNA-Seq: RNA sequencing
SC-islet: Stem cell-derived islet
