# Supplementary material for: The promise of stem cell-derived islet replacement therapy
Source: Diabetologia. 2021 Jan 16;64(5):1030–6. doi: 10.1007/s00125-020-05367-2 (PMC8012315; doi:10.1007/s00125-020-05367-2)
Supplement: Supplementary file 1 — (PPTX 1.01 mb) [file 125_2020_5367_MOESM1_ESM.pptx]

## Slide 1
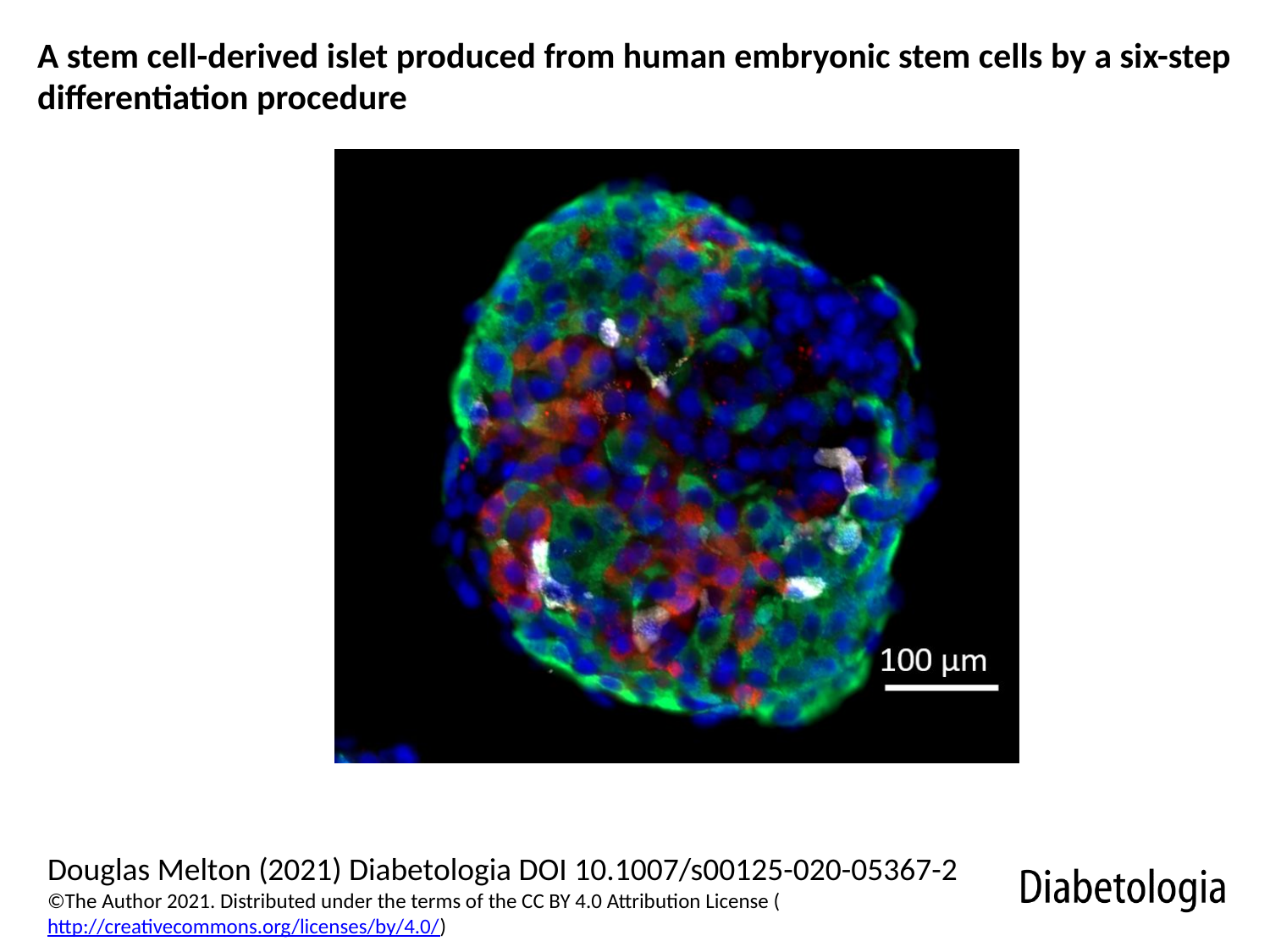

A stem cell-derived islet produced from human embryonic stem cells by a six-step differentiation procedure
Douglas Melton (2021) Diabetologia DOI 10.1007/s00125-020-05367-2
©The Author 2021. Distributed under the terms of the CC BY 4.0 Attribution License (http://creativecommons.org/licenses/by/4.0/)

## Slide 2
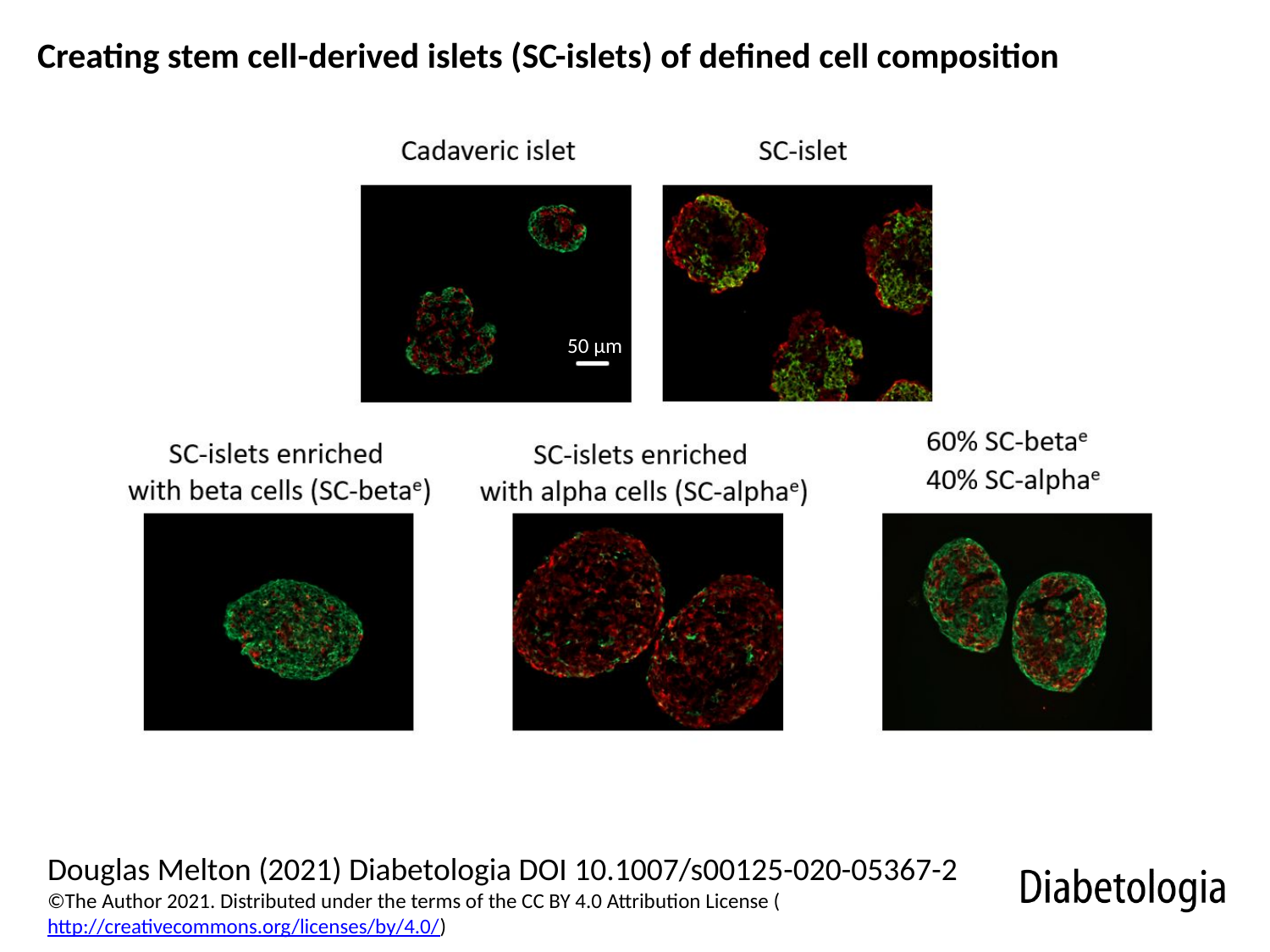

Creating stem cell-derived islets (SC-islets) of defined cell composition
50 µm
Douglas Melton (2021) Diabetologia DOI 10.1007/s00125-020-05367-2
©The Author 2021. Distributed under the terms of the CC BY 4.0 Attribution License (http://creativecommons.org/licenses/by/4.0/)

## Slide 3
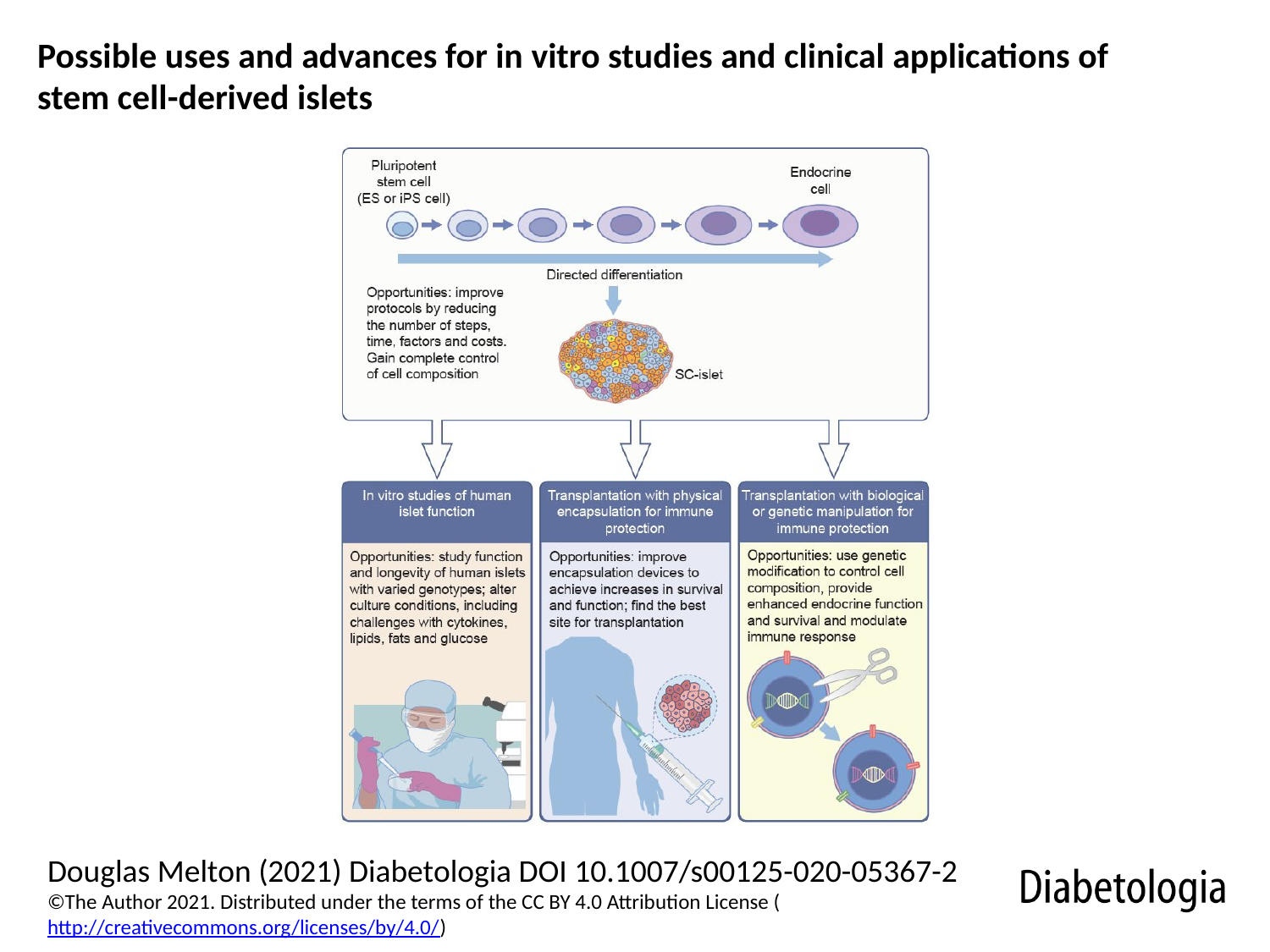

Possible uses and advances for in vitro studies and clinical applications of stem cell-derived islets
Douglas Melton (2021) Diabetologia DOI 10.1007/s00125-020-05367-2
©The Author 2021. Distributed under the terms of the CC BY 4.0 Attribution License (http://creativecommons.org/licenses/by/4.0/)
